# Supplementary material for: Electrical switching of Ising-superconducting nonreciprocity for quantum neuronal transistor
Source: Nat Commun. 2024 Jun 10;15:4953. doi: 10.1038/s41467-024-48882-1 (PMC11164936; doi:10.1038/s41467-024-48882-1)
Supplement: Supplementary file 1 — Supplementary Materials [file 41467_2024_48882_MOESM1_ESM.docx]

Supplementary Information

Electrical switching of Ising-superconducting nonreciprocity for quantum neuronal transistor

Junlin Xiong^1†^, Jiao Xie^1†^, Bin Cheng^2*^, Yudi Dai^1^, Xinyu Cui^1^, Lizheng Wang^1^, Zenglin Liu^1^, Ji Zhou^1^, Naizhou Wang^3^, Xianghan Xu^4^, Xianhui Chen^3^, Sang-Wook Cheong^4^, Shi-Jun Liang^1*^, Feng Miao^1*^

^1^Institute of Brain-Inspired Intelligence, National Laboratory of Solid State Microstructures, School of Physics, Collaborative Innovation Center of Advanced Microstructures, Nanjing University, Nanjing 210093, China

^2^Institute of Interdisciplinary Physical Sciences, School of Science, Nanjing University of Science and Technology, Nanjing 210094, China

^3^Hefei National Laboratory for Physical Science at Microscale and Department of Physics and Key Laboratory of Strongly Coupled Quantum Matter Physics, University of Science and Technology of China, Hefei, Anhui 230026, China

^4^Center for Quantum Materials Synthesis and Department of Physics and Astronomy, Rutgers, The State University of New Jersey, Piscataway, NJ, 08854, USA

† Contribute equally to this work.

*Correspondence email: bincheng@njust.edu.cn; [sjliang@nju.edu.cn](mailto:sjliang@nju.edu.cn);

[miao@nju.edu.cn](mailto:miao@nju.edu.cn)

**I. Thickness of the PAIS device determined by atomic force microscope**

**II. Anomalous hall effect for different temperatures**

**III. Characterization of magnetic field dependence of superconductivity**

**IV. Characterization of superconductivity with magnetic proximity**

**V. Superconducting diode effect for different magnetization states under larger external magnetic fields**

**VI. Second harmonic measurement for magnetization “UP” and “DOWN” state**

**VII. Nonreciprocal superconducting transport at different temperatures**

**VIII. Schematic of the mechanism for field-free electrical switching of perpendicular magnetization.**

**IX. Reproducibility of the electrically switchable superconducting nonreciprocity and function of quantum neuronal transistor**

**X. Comparison of magnetoresistance (MR) and resistance-area (RA) product between this work and previous literatures**

**XI. Symmetry mechanism of electrically switchable superconducting nonreciprocity**

**XII. Second harmonic generation measurements to determine crystallographic orientation**

**I. Thickness of the PAIS device determined by atomic force microscope**

**
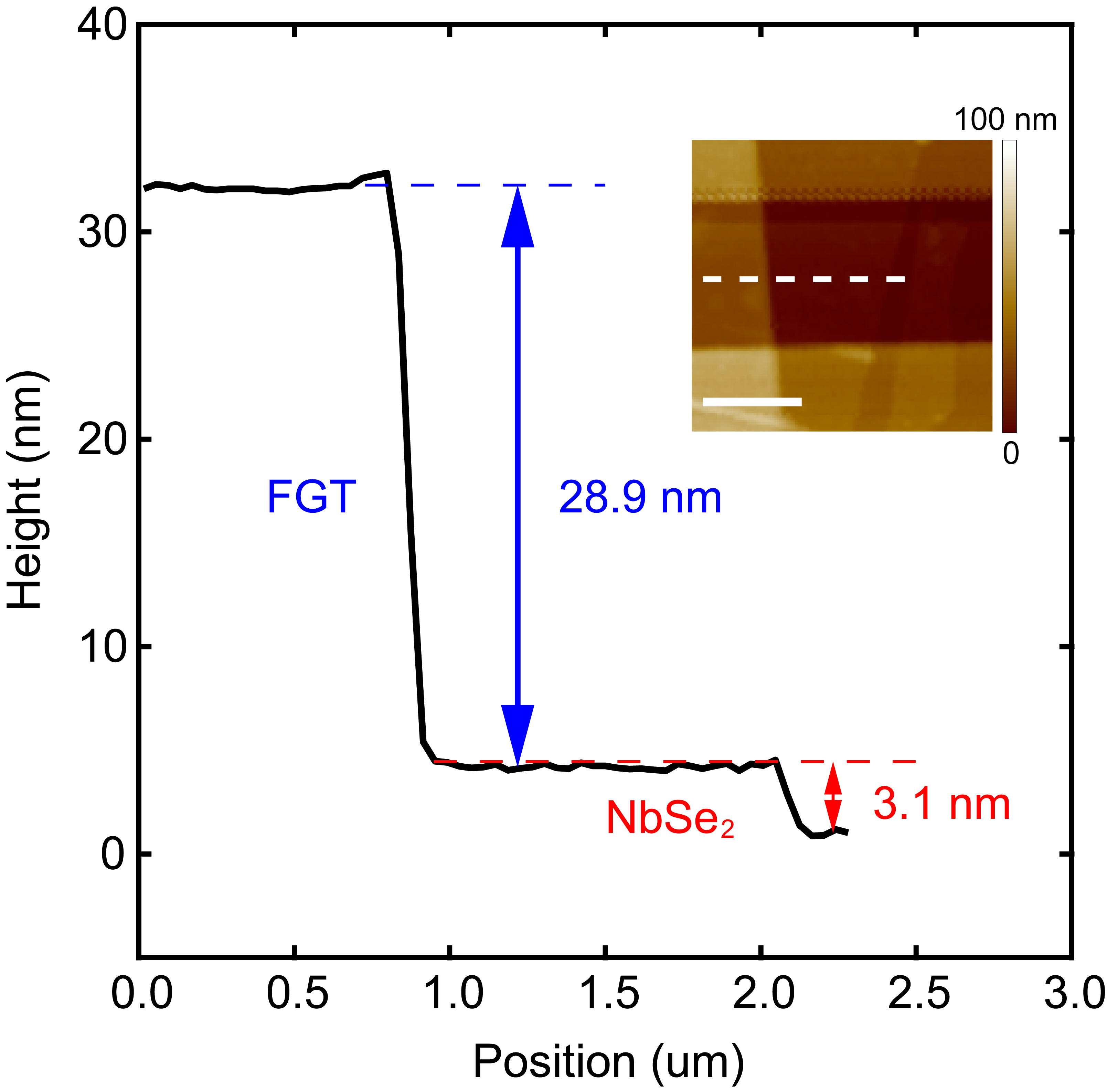
**

**Supplementary Fig. 1.** Height profile of the PAIS device determined by atomic force microscope. The FGT and $\mathrm{NbSe}_{2}$ flakes are about 28.9 nm and 3.1 nm in thickness, respectively, as determined by atomic force microscope. AFM scanning position is represented by a white line in AFM image, shown in the inset. Scale bar is 1 μm.

**II. Anomalous hall effect for different temperatures**

**
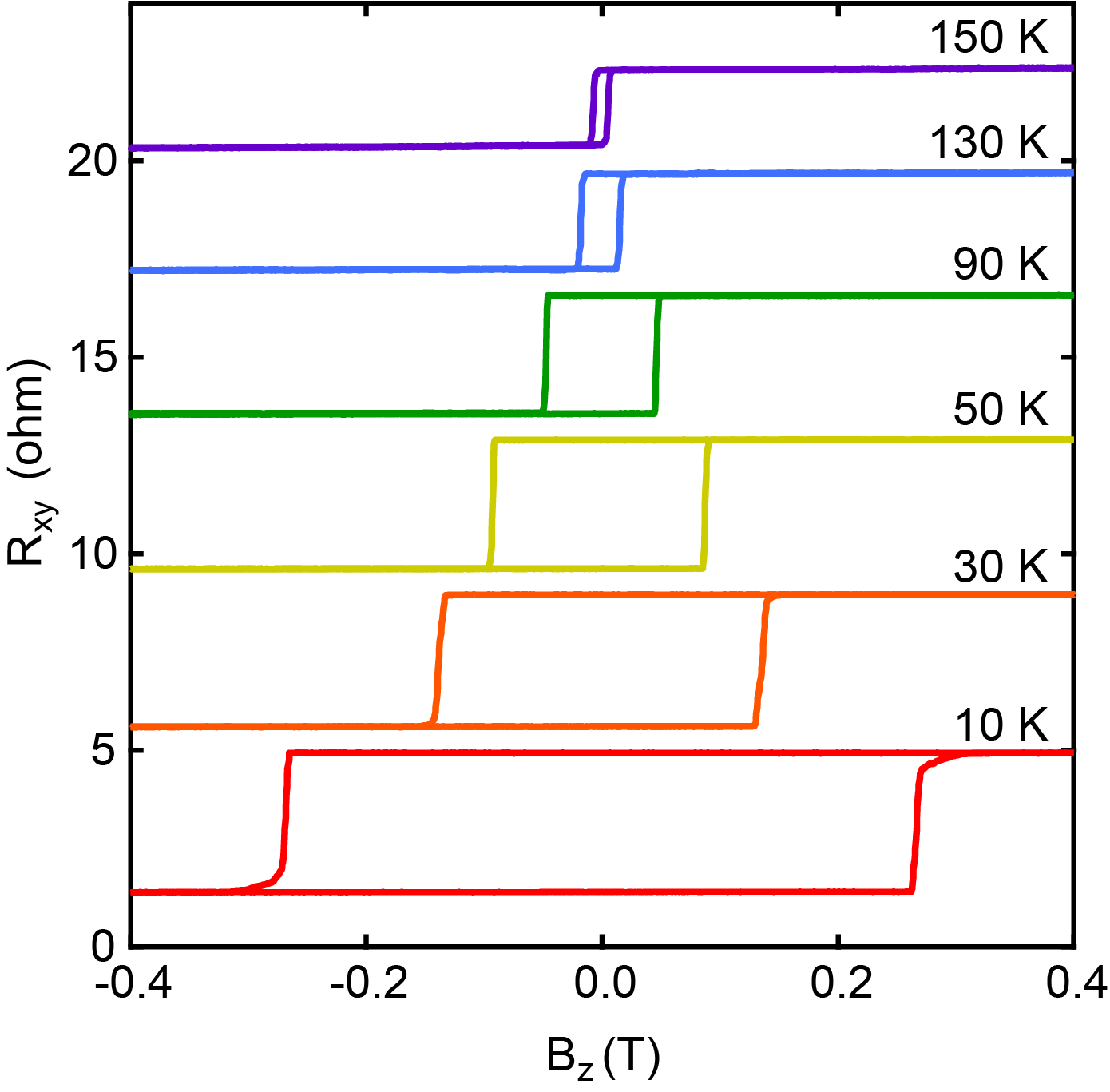
**

**Supplementary Fig. 2.** Hall resistance as a function of the perpendicular magnetic field for temperatures ranging from 10 to 150 K. The square-shaped hysteresis loop appearing at the low temperature indicates that the PAIS material possesses the perpendicular magnetic anisotropy.

**III. Characterization of magnetic field dependence of superconductivity**

We characterized the transport properties of a PAIS device for different magnetic fields and temperatures (see Supplementary Fig.3a-b). As the applied perpendicular magnetic field or temperature increases, the superconductivity is suppressed.


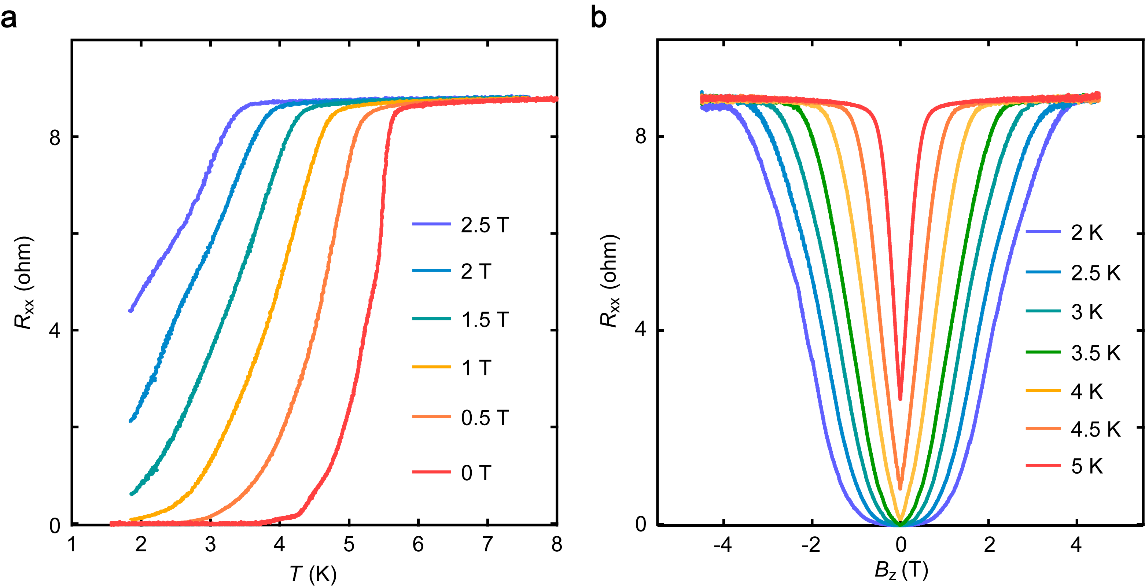


**Supplementary Fig. 3.** Magnetic field and temperature dependence of the PAIS device resistance. **a**, Temperature dependence of the device resistance for magnetic fields ranging from 0 to 2.5 T. **b**, Magnetic field dependence of the device resistance for temperatures ranging from 2 to 5 K. This PAIS device consisting of seven-layer NbSe_2_ flake was measured by applying an electrical current of 0.5 μA.

**IV. Characterization of superconductivity with magnetic proximity**

We measured the temperature dependent resistances of the NbSe_2_, FGT and NbSe_2_/FGT samples, with the corresponding results shown in Supplementary Fig. 4. The superconducting transition temperature *T*_c_ of the NbSe_2_/FGT heterostructure with 7-layer (7L) NbSe_2_ is 4.8 K and lower than that of the 7L-NbSe_2_ sample (6.1 K), which can be attributed to the pair-breaking effect induced by the magnetization of FGT.


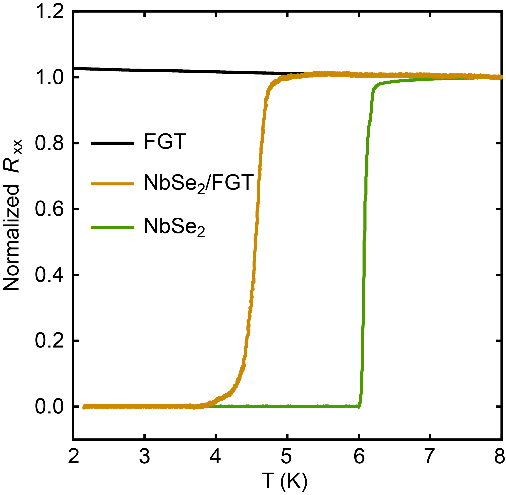


**Supplementary Fig. 4.** Temperature dependent normalized longitudinal resistances (defined as *R*_xx_(T)/*R*_xx_(8K)) of the NbSe_2_ (green line), FGT (black line) and NbSe_2_/FGT (orange line) samples.

**V. Superconducting diode effect for different magnetization states under larger external magnetic fields**


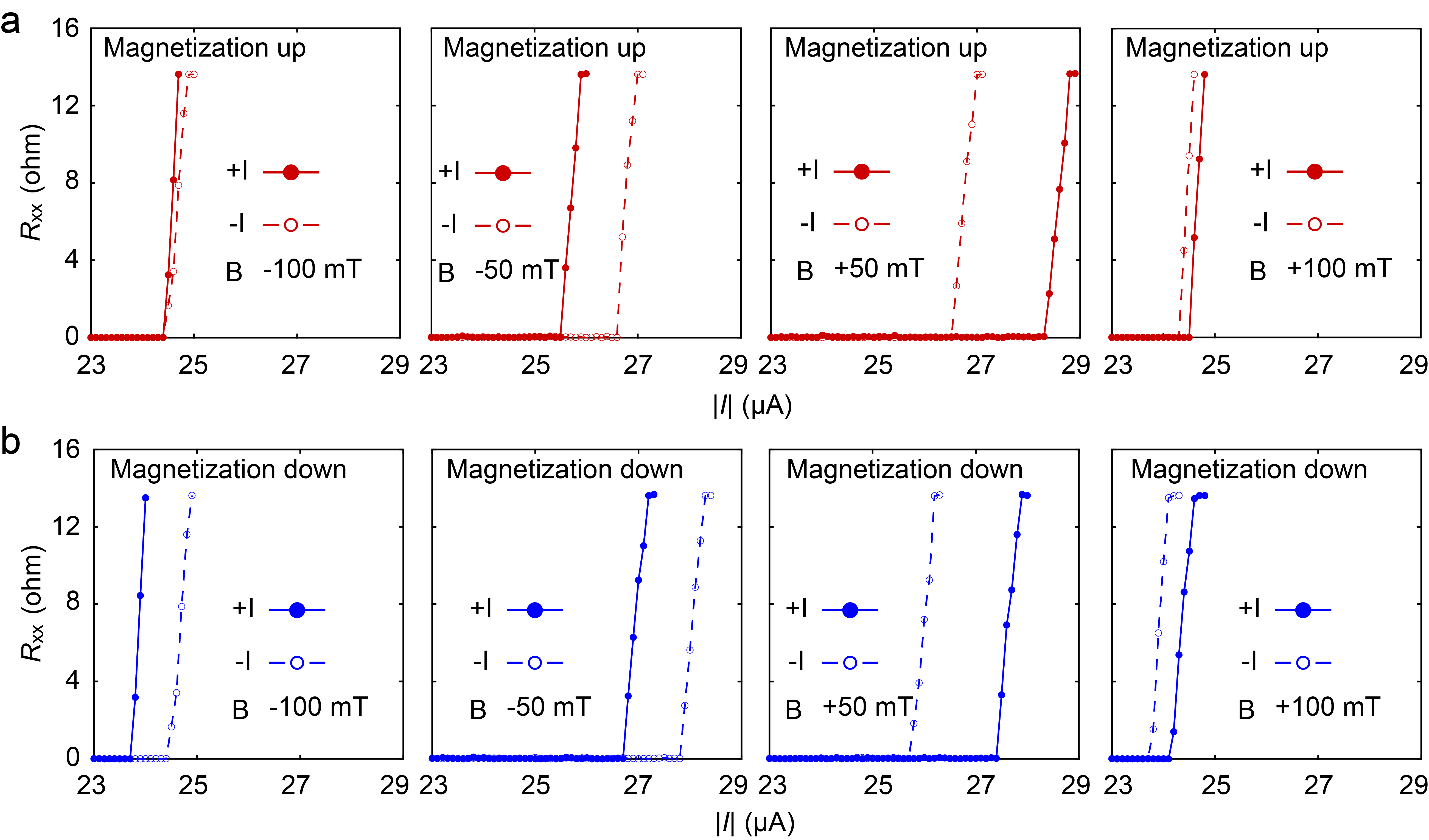


**Supplementary Fig. 5.** **a,** Current dependences of the resistance under different perpendicular magnetic fields B=-100 mT, -50 mT, 50 mT, and 100 mT for both positive and negative currents at 1.6 K when the magnetization is set as “UP” state. **b,** Current dependences of the resistance under different perpendicular magnetic fields B=-100 mT, -50 mT, 50 mT, and 100 mT for both positive and negative currents at 1.6 K when the magnetization is set as “DOWN” state.

**VI. Second harmonic measurement for magnetization “UP” and “DOWN” state**

**
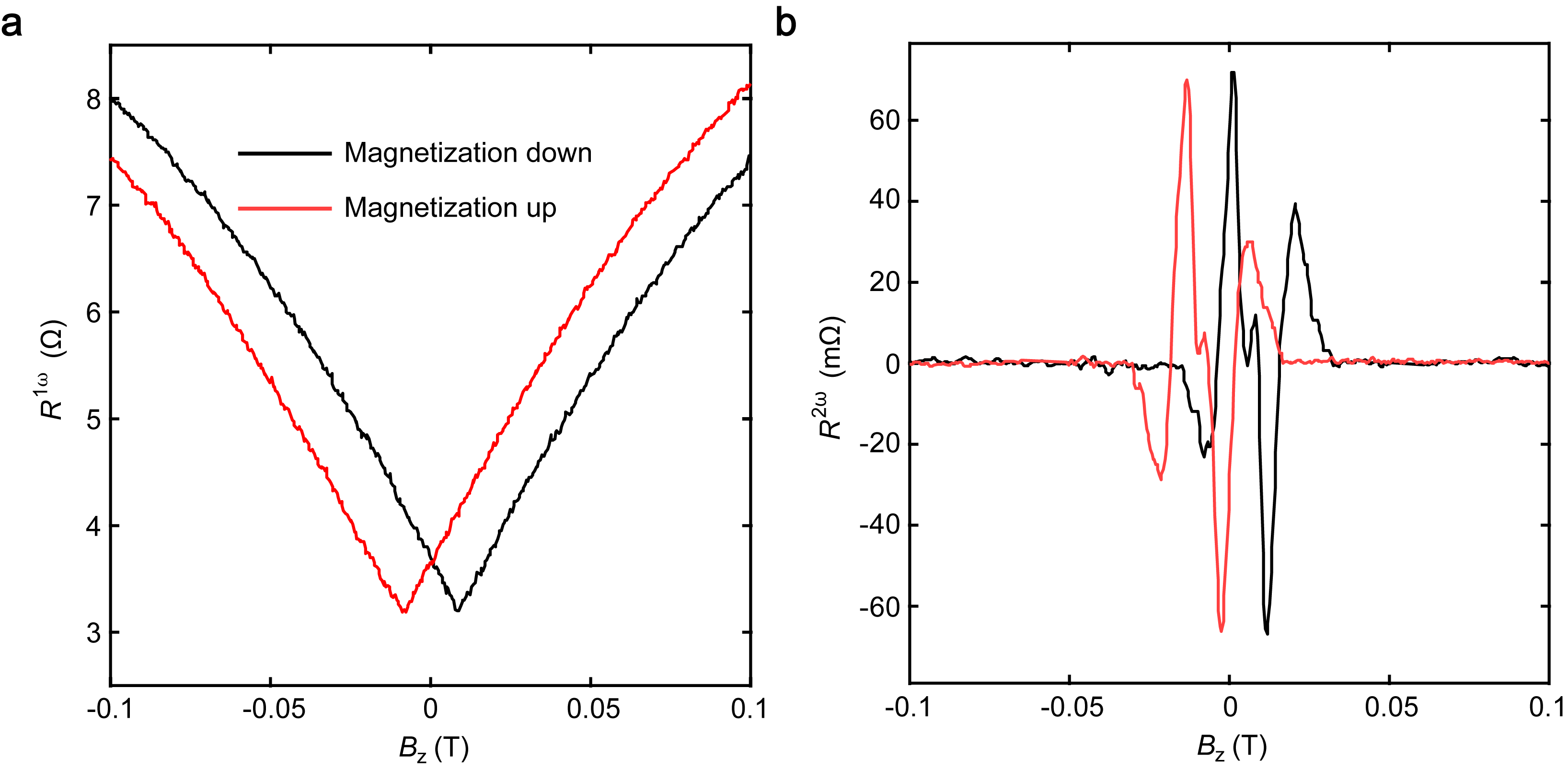
**

**Supplementary Fig. 6.** Second harmonic measurement for magnetization “UP” and “DOWN” state. **a,** The linear resistance $R^{\omega}$ as a function of the magnetic field for distinct magnetization states. **b,** The second-harmonic resistance $R^{2\omega}$ as a function of the magnetic field for distinct magnetization states. The red and black lines represent the cases for the magnetization “UP” and “DOWN” states, respectively.

**VII. Nonreciprocal superconducting transport at different temperatures**

**
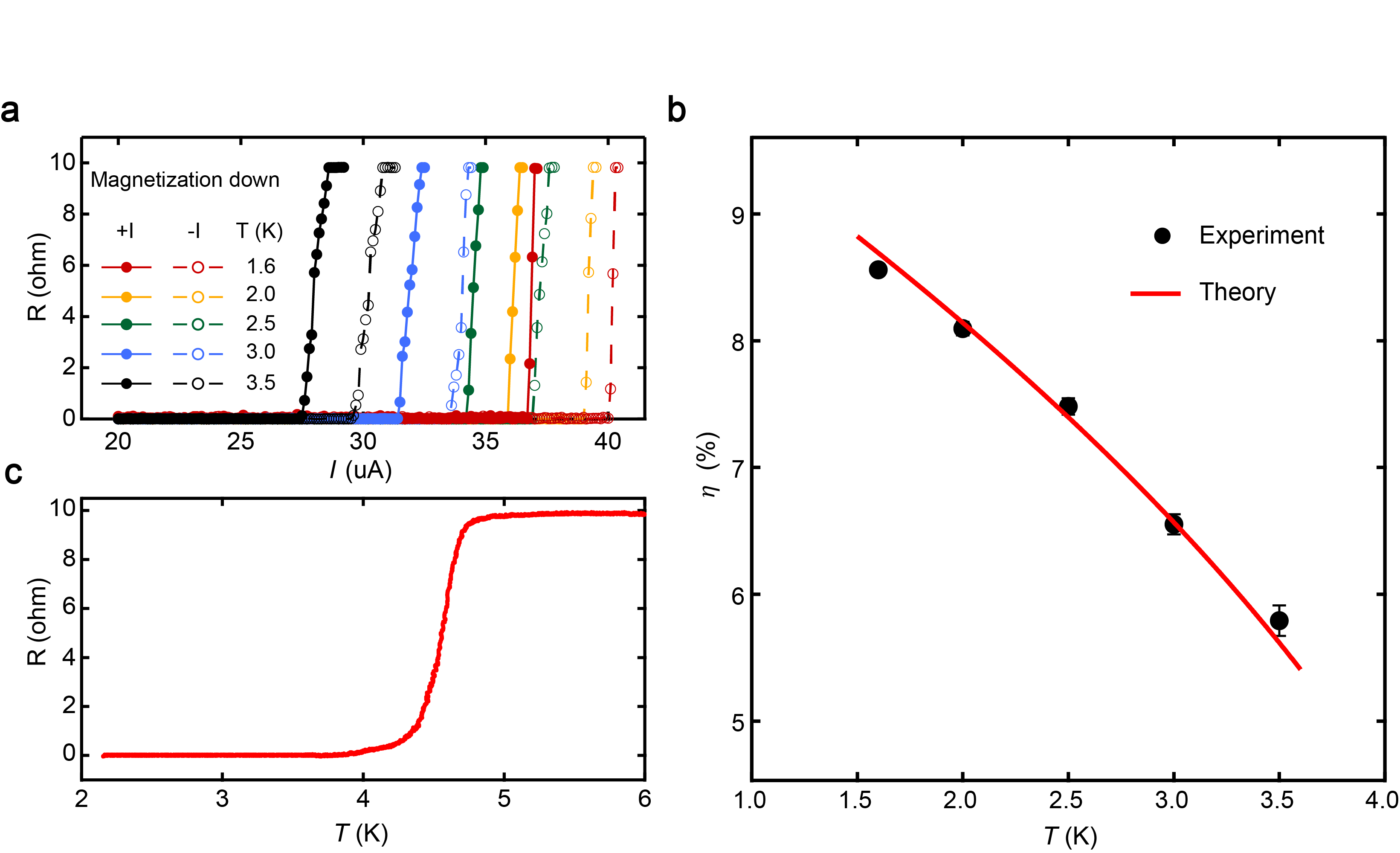
**

**Supplementary Fig. 7.** Nonreciprocal superconducting transport at different temperatures when the magnetization is fixed as “DOWN” state. **a,** Current dependences of the resistance for both positive and negative currents at different temperatures ranging from 1.6 K to 3.5 K. **b,** The zero-field nonreciprocal efficiency $\eta$ as a function of the temperature when the magnetization is fixed as “DOWN” state. **c,** The temperature dependence of the device resistance shows superconducting temperature *T*_c_ ≈ 4.8 K.

**VIII. Schematic of the mechanism for field-free electrical switching of perpendicular magnetization.**


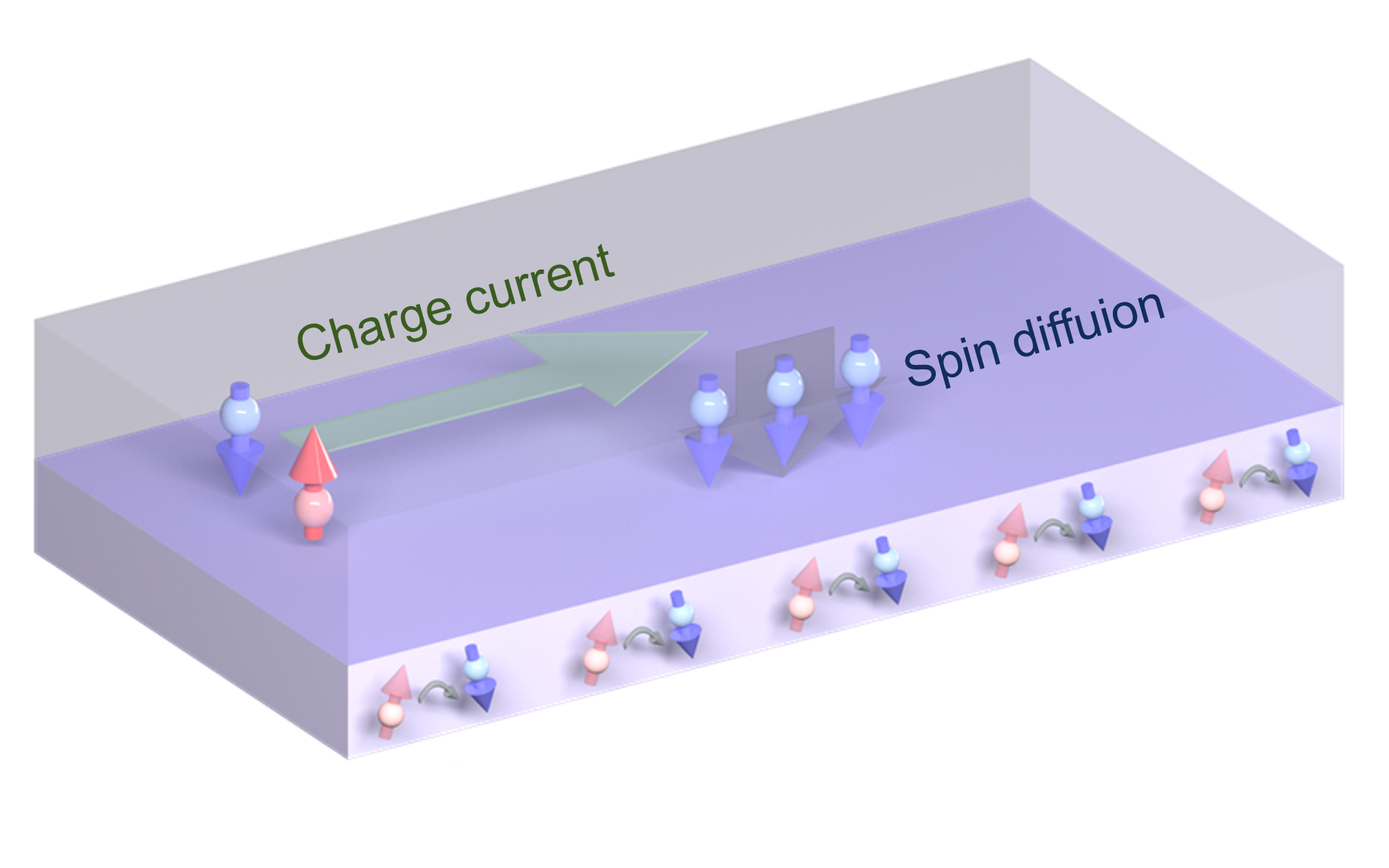


**Supplementary Fig. 8.** Schematic of the mechanism for field-free electrical switching of perpendicular magnetization. The green arrow represents the electron flow (along the x direction), which generates out-of-plane spin polarization accumulating at the interface and diffusing into the magnetic layer. The current-induced spin polarization pointing in the z (-z) direction is indicated by the blue (red) arrows in the top layer. The red and blue arrows in the bottom layer represent the initial and resulting magnetization states.

**IX. Reproducibility of the electrically switchable superconducting nonreciprocity and function of quantum neuronal transistor**

We fabricated three different devices with odd-layer and even-layer NbSe_2_, respectively, and all the devices have the similar behaviors of electrically switchable superconducting nonreciprocity and function of quantum neuronal transistor, as shown in Supplementary Fig. 9-11.


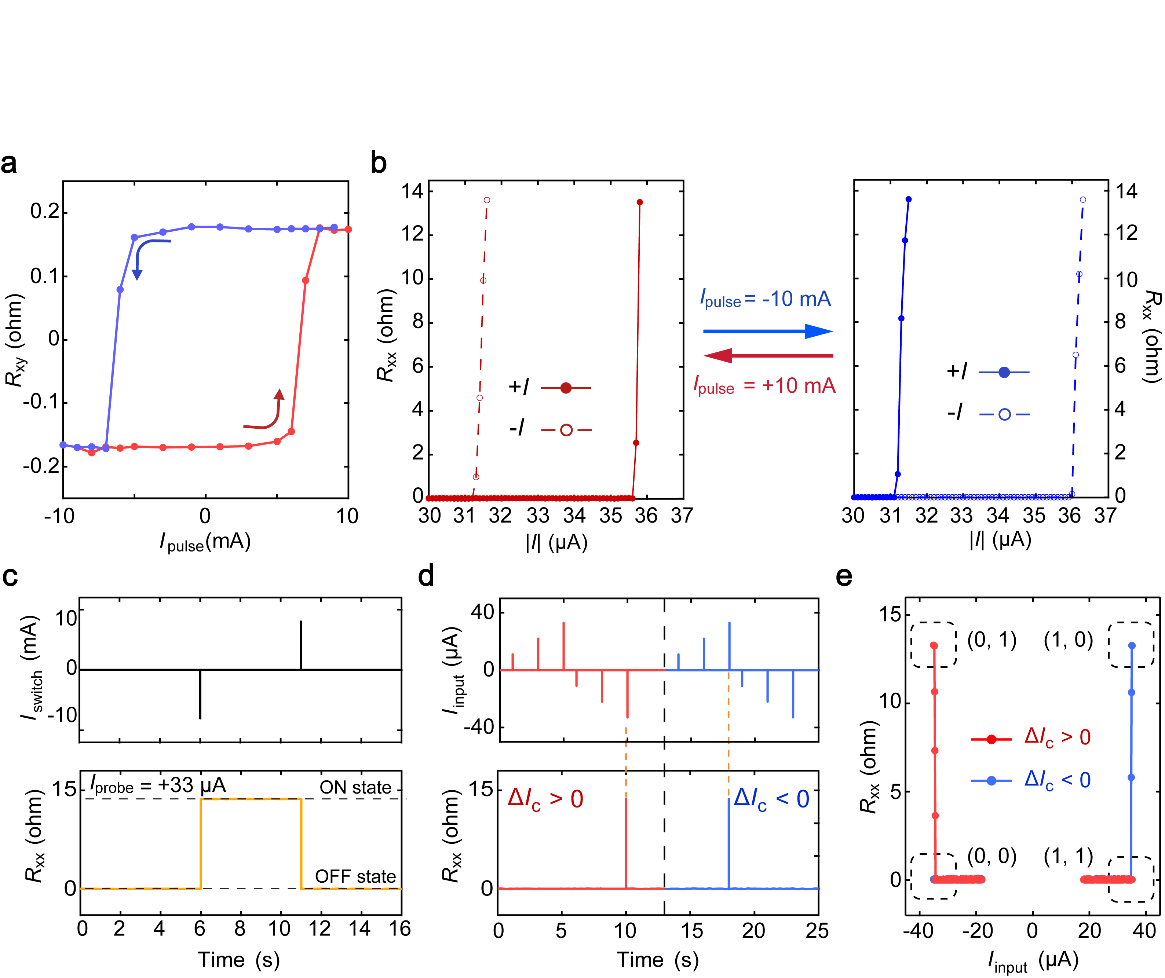


**Supplementary Fig. 9.** Electrically switchable superconducting nonreciprocity and functionality of nonreciprocal neural transistor in a five-layer device. **a**, Current-induced magnetization switching at zero field at 1.6 K. **b**, electrically switchable nonreciprocal superconducting transport. **c**, Deterministic switching by a series of current pulses applied in the device. The width and magnitude of the current pulses are 200 μs and 8 mA, respectively. The resistance is measured by using a small d.c. excitation current of +33 μA. **d**, The responses of spike to the input current pulses for the polarity “+” and “-” states. **e**, The XOR function in the nonreciprocal neural transistor. The dashed boxes represent the logic state values for input and polarity combinations (0,1), (1,1), (0,0) and (1,0), respectively.


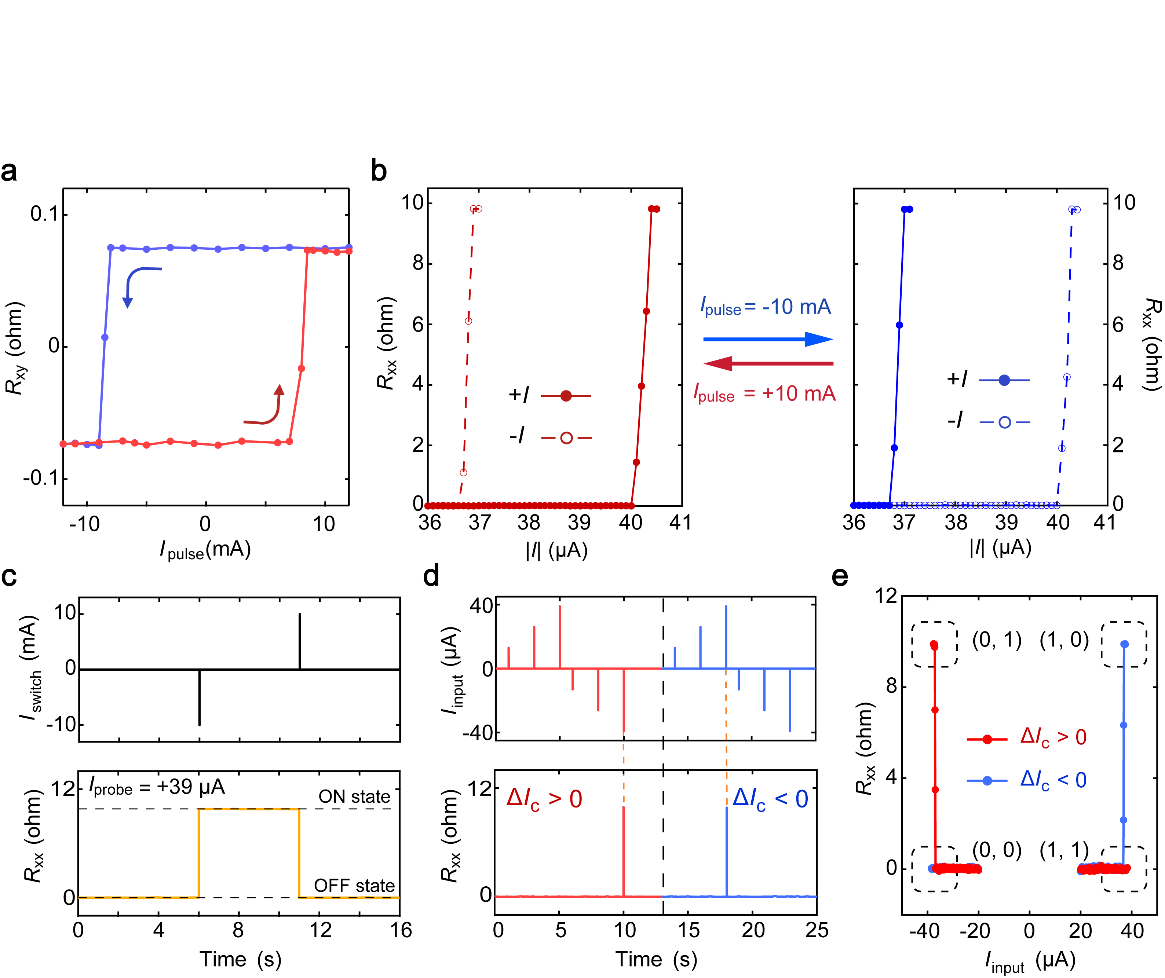


**Supplementary Fig. 10.** Electrically switchable superconducting nonreciprocity and functionality of nonreciprocal neural transistor in a seven-layer device. **a**, Current-induced magnetization switching at zero field at 1.6 K. **b**, electrically switchable nonreciprocal superconducting transport. **c**, Deterministic switching by a series of current pulses applied in the device. The width and magnitude of the current pulses are 200 μs and 10 mA, respectively. The resistance is measured by using a small d.c. excitation current of +39 μA. **d**, The responses of spike to the input current pulses for the polarity “+” and “-” states. **e**, The XOR function in the nonreciprocal neural transistor. The dashed boxes represent the logic state values for input and polarity combinations (0,1), (1,1), (0,0) and (1,0), respectively.


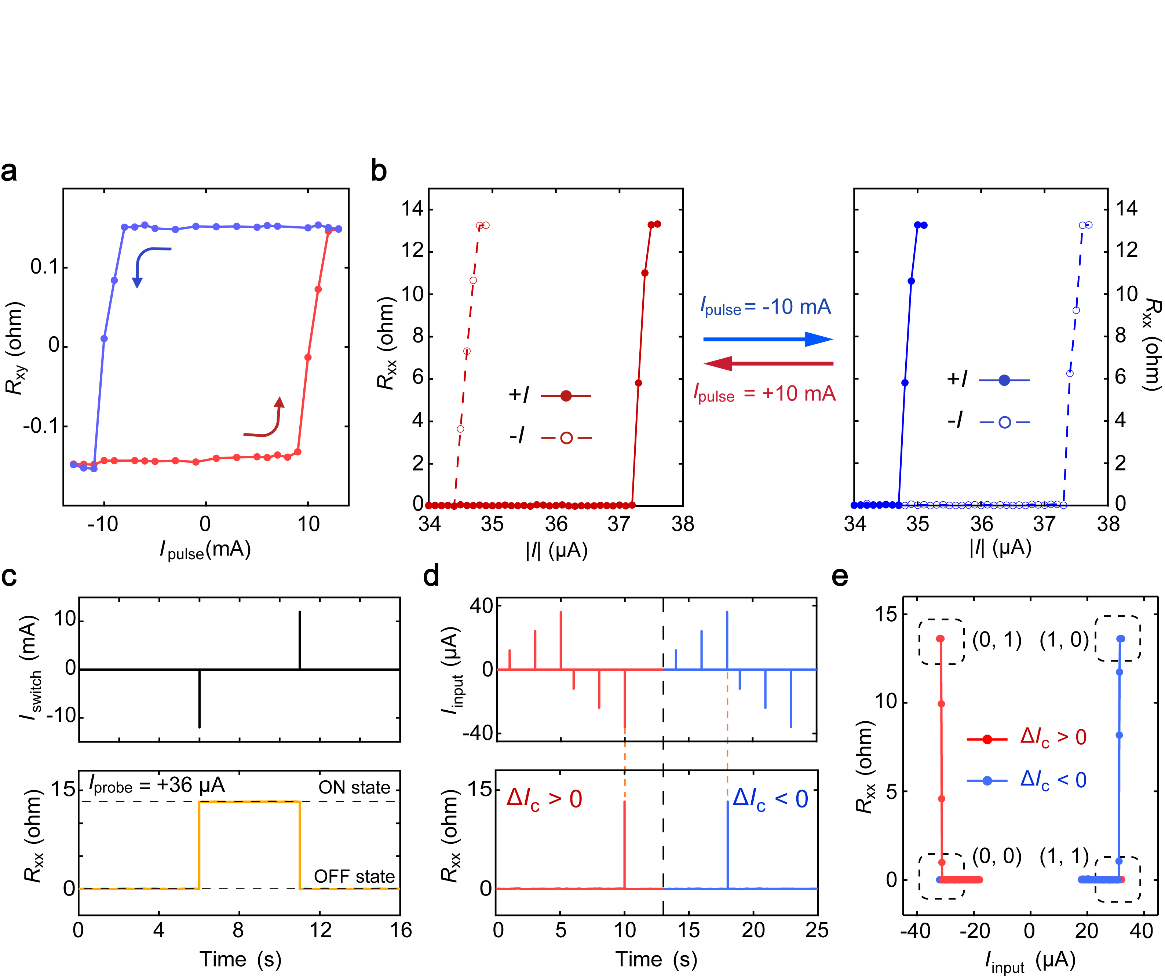


**Supplementary Fig. 11.** Electrically switchable superconducting nonreciprocity and functionality of nonreciprocal neural transistor in a six-layer device. **a**, Current-induced magnetization switching at zero field at 1.6 K. **b**, electrically switchable nonreciprocal superconducting transport. **c**, Deterministic switching by a series of current pulses applied in the device. The width and magnitude of the current pulses are 200 μs and 12 mA, respectively. The resistance is measured by using a small d.c. excitation current of +36 μA. **d**, The responses of spike to the input current pulses for the polarity “+” and “-” states. **e**, The XOR function in the nonreciprocal neural transistor. The dashed boxes represent the logic state values for input and polarity combinations (0,1), (1,1), (0,0) and (1,0), respectively.

**X. Comparison of magnetoresistance (MR) and resistance-area (RA) product between this work and previous literatures**


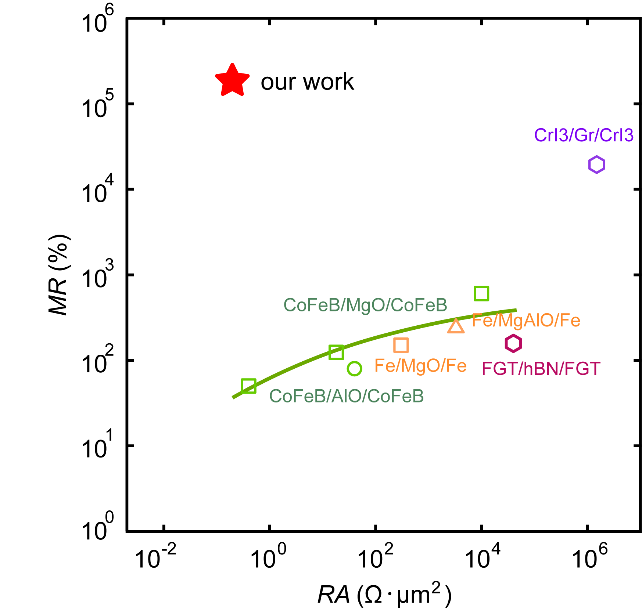


**Supplementary Fig. 12.** Comparison of magnetoresistance (MR) and resistance-area (RA) product between this work and previous literatures. Relationships between MR and RA of magnetic tunnel junctions (MTJs) with MgO-based (square)^1-3^, AlO-based (circle)^4^, MgAlO-based (triangle)^5^ and van der Waals (vdW) material-based (hexagon)^6,7^ barrier are obtained from previous literatures. Different colors are used to distinguish different magnetic material components. The results from our work are denoted by the star symbol. The green solid line represents the tradeoff between MR and RA in the conventional MTJ. The RA of MTJ increases dramatically with the promotion of magnetoresistance.

**XI. Symmetry mechanism of electrically switchable superconducting nonreciprocity**

With the ubiquitous strain and lattice mismatch at the vdW interface and/or trigonal warping effect, lowering of *C*_3_ symmetry breaks mirror symmetry *M*_y_. On the one hand, the ubiquitous strain and lattice mismatch would break mirror symmetry *M*_y_ to generate the in-plane electric polarization *P*_y_, but also break mirror symmetry *M*_x_. In this way, the magneto-toroidal nonreciprocal directional dichroism (NDD) effect would be maximal due to an optimized nonreciprocal term $\hat{\mathbf{y}}\cdot(\mathbf{M}_{z}\boldsymbol{\times}\mathbf{I}_{x})$ and a maximum valley magnetization $\mathbf{P}_{y}\times\mathbf{I}_{x}$. On the other hand, with the current along the zigzag direction, the Hamiltonian with the trigonal warping effect, i.e., $H=\left( \frac{k_{x}^{2}+k_{y}^{2}}{2m}-\mu\right)\sigma_{0}+\lambda_{I}k_{x}\left( k_{x}^{2}-3k_{y}^{2} \right)\sigma_{z},$would break the mirror symmetry $M_{y}$ since $\mathcal{M}_{y}H\mathcal{M}_{y}^{-1}\neq H.$In contrast, the mirror symmetry $M_{x}$ is preserved since $\mathcal{M}_{x}H\mathcal{M}_{x}^{-1}=H$. With the assist of the magnetization proximity, the $M_{y}$ symmetry breaking allows to produce a finite momentum of Cooper pairs ($q_{x}=\frac{2\alpha_{0}}{3\alpha_{3}}\left| \mathbf{M}_{z}\times\hat{\boldsymbol{y}} \right|$), leading to superconducting nonreciprocity. Here, $\alpha_{0}$ is $A_{0}(T-T_{c})$ with the constant $A_{0}>0$, and $\alpha_{3}$ is determined by Fermi surface properties of the Ising superconductor considering the trigonal warping effect. In addition, the current-induced z spin polarization that contributes to magnetization switching also requires this symmetry breaking, as shown in Supplementary Fig. 13. Therefore, as the current flows along the zigzag direction, these two effects (i.e., strain and lattice mismatch at the vdW interface, and trigonal warping effect) can coexist and simultaneously contribute to the electrically switchable superconducting nonreciprocity.


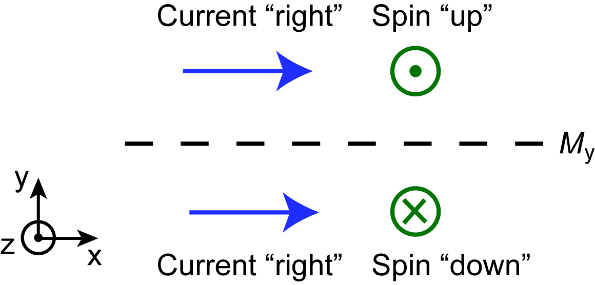


**Supplementary Fig. 13.** Current-induced z spin polarization with the *M*_y_ symmetry preserved. The current in the x direction is unchanged while the spin in the z direction will be reversed under My symmetry operation.

**XII. Second harmonic generation measurements to determine crystallographic orientation**


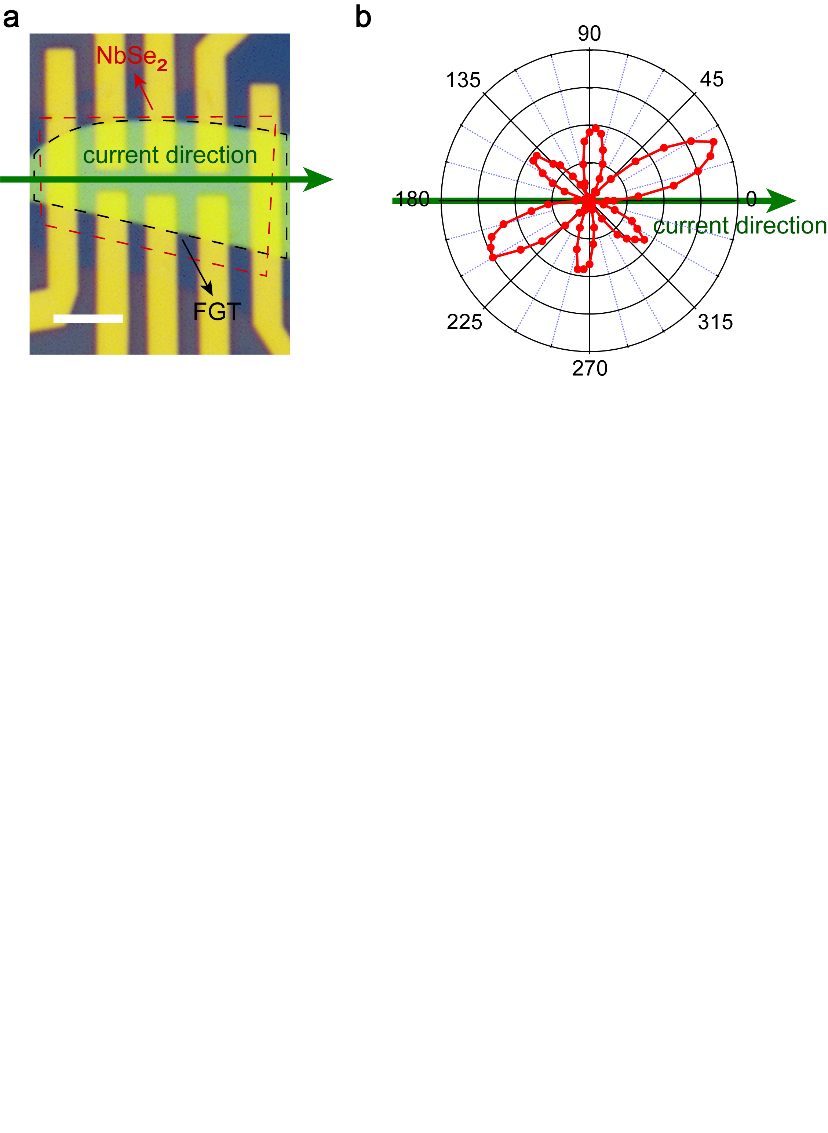


**Supplementary Fig. 14.** Second harmonic generation measurements to determine the relationship between the current direction and crystallographic orientation. **a**, Optical image of a PAIS device. The scale bar is 3 μm. **b**, Polar plot of the second harmonic generation (SHG) signal. The green arrow represents the current direction.

**Supplementary References:**

1 Parkin, S. S. P. *et al.* Giant tunnelling magnetoresistance at room temperature with MgO (100) tunnel barriers. *Nat. Mater.* **3**, 862-867 (2004).

2 Yuasa, S. *et al.* Giant room-temperature magnetoresistance in single-crystal Fe/MgO/Fe magnetic tunnel junctions. *Nat. Mater.* **3**, 868-871 (2004).

3 Ikeda, S. *et al.* Tunnel magnetoresistance of 604% at 300K by suppression of Ta diffusion in CoFeB∕MgO∕CoFeB pseudo-spin-valves annealed at high temperature. *Appl. Phys. Lett.* **93**, 082508 (2008).

4 Wei, H. X. *et al.* 80% tunneling magnetoresistance at room temperature for thin Al–O barrier magnetic tunnel junction with CoFeB as free and reference layers. *J. Appl. Phys.* **101**, 09B501 (2007).

5 Sukegawa, H. *et al.* Tunnel magnetoresistance with improved bias voltage dependence in lattice-matched Fe/spinel MgAl_2_O_4_/Fe(001) junctions. *Appl. Phys. Lett.* **96**, 212505 (2010).

6 Song, T. *et al.* Giant tunneling magnetoresistance in spin-filter van der Waals heterostructures. *Science* **360**, 1214-1218 (2018).

7 Wang, Z. *et al.* Tunneling Spin Valves Based on Fe_3_GeTe_2_/hBN/Fe_3_GeTe_2_ van der Waals Heterostructures. *Nano Lett.* **18**, 4303-4308 (2018).
